# A Comparative Study on Phenolic Content, Antioxidant Activity and Anti-Inflammatory Capacity of Aqueous and Ethanolic Extracts of Sorghum in Lipopolysaccharide-Induced RAW 264.7 Macrophages

**DOI:** 10.3390/antiox9121297

**Published:** 2020-12-18

**Authors:** Shan Hong, Philipus Pangloli, Ramasamy Perumal, Sarah Cox, Leela E. Noronha, Vermont P Dia, Dmitriy Smolensky

**Affiliations:** 1 Department of Food Science, The University of Tennessee Institute of Agriculture, Knoxville, TN 37996, USA; shong19@vols.utk.edu (S.H.); ppanglol@utk.edu (P.P.); 2 Agricultural Research Center, Kansas State University, Hays, KS 67601, USA; Perumal@ksu.edu; 3 Center for Grain and Animal Health Research, USDA-ARS, Manhattan, KS 66502, USA; sarah.cox@usda.gov (S.C.); Leela.noronha@usda.gov (L.E.N.)

**Keywords:** sorghum, phenolics, tannins, extraction, antioxidant, anti-inflammatory, RAW 264.7

## Abstract

Sorghum is an important cereal with diverse phenolic compounds that have potential health promoting benefits. The current study comparatively characterized the phenolic contents of two novel black-seeded sorghum lines (SC84 and PI570481) using different extraction systems (water, ethanol and their acidified counterparts) and evaluated their antioxidant and anti-inflammatory activities. Phenolic compositions were determined by spectrophotometric assays and HPLC analysis. Antioxidant activities were assessed by radical scavenging effects on nitric oxide (NO) and 2,2-diphenyl-1-picrylhydrazyl (DPPH) free radicals, and the oxygen radical absorbance capacity (ORAC). Anti-inflammatory capacity was estimated by measuring levels of pro-inflammatory markers produced by lipopolysaccharide (LPS)-induced RAW 264.7 macrophages. Results showed that effects of solvent types and HCl on extraction efficiency differed among phenolic compounds and sorghum samples. Tannins were the most dominant polyphenols in the studied extracts (11.11–136.11 mg epicatechin equivalent/g sorghum). Sorghum extracts exerted more potent scavenging activity on DPPH than NO radicals. In LPS-activated RAW 264.7 cells, sorghum extracts dose-dependently inhibited the production of NO, interleukin-6 (IL-6), and intracellular reactive oxygen species (ROS), with ethanolic extracts showing greater anti-inflammatory activity. Positive correlations were noted between tannin content and DPPH radical scavenging activity, and anti-inflammatory capacity. These results suggest the potential role of tannin-rich sorghum extracts against inflammation and associated diseases.

## 1. Introduction

Sorghum, a member of the Poaceae grass family, is one of the leading dryland crops in the world, appearing to be the fifth most produced cereal next to rice, wheat, barley, and corn [1,2]. It has been widely cultivated worldwide, serving as food, feed, and fuel [3]. The main components of sorghum are polysaccharides (starch and non-starch), proteins, and lipids [1,2]. More importantly, sorghum is an excellent source of bioactive phenolic compounds that are more abundant in both content and diversity compared to other major cereal crops [1,2]. Almost all classes of phenolic compounds have been identified in sorghum, with simple phenolic acids, flavonoids, and tannins being the dominant groups [1,2]. Sorghum anthocyanins are unique due to the absence of a hydroxyl group at position C-3, thus the name 3-deoxyanthocyanidins, which comprise a rare subclass of anthocyanins [4]. It has been reported that sorghum 3-deoxyanthocyanidins, composed mainly of luteolinidin and apigeninidin, are more stable in slightly acidic conditions in the presence of sulfites [5] and are more resistant to the photo-bleaching effects of ascorbic acid than the corresponding 3-oxygenated anthocyanin counterparts [6], thus supporting the role of 3-deoxyanthocyanidin as a source of natural pigment. In addition, sorghum tannins, composed of oligomers or polymers of mainly catechins (flavan-3-ol or flavan-3,4-diol) with high molecular weight and a high degree of polymerization (DP), are rarely found among other major cereals [7].

The unique phenolic composition of sorghum makes it a promising food source with a number of potential health promoting benefits. Dia et al. reported the capability of sorghum phenolic extract to suppress interleukin (IL)-1β and IL-18 secretion in lipopolysaccharide (LPS)-primed and adenosine triphosphate (ATP)-activated THP-1 human macrophages, associated with a reduction in caspase-1 [8]. Previous studies have shown a wide range of potential biological activities of sorghum 3-deoxyanthocyanidins, including anti-proliferative and pro-apoptotic effects against colon cancer cells [9] and reducing oxidative stress via increasing the NADH:quinone oxyreductase (NQO) ratio [10]. Smolensky et al. demonstrated the versatile biological roles of phenolic extracts of PI570481, a novel high polyphenol sorghum type, including anti-cancer and anti-microbial effects [11,12,13,14]. In addition to their powerful antioxidant ability [15], sorghum-condensed tannins have shown great inhibitory activities against digestive enzymes, which may partially contribute to the anti-diabetic capacity of sorghum [16]. Given the high health potential, sorghum has attracted great attention for decades.

Oxidative stress, referring to the imbalance of the excessive production of free radicals and antioxidants, is highly implicated in various non-communicable diseases such as inflammation, cancer and cardiovascular disease [17,18]. Inflammation is a defensive response of the immune system against external and internal stimuli that can cause infection and injury [19]. However, uncontrolled and chronic inflammation has been associated with the development of several diseases including obesity [20], type 2 diabetes [21], and several cancers [22]. As innate cells, macrophages are recruited to inflammatory sites, activated, and release cascades of inflammatory molecules, including nitric oxide (NO) and IL-6 under the stimulation of LPS, a well-known endotoxin from Gram-negative bacteria [23]. NO is a gaseous free radical regulated by inducible nitric oxide synthase (iNOS). Excessive NO can cause DNA damage, mutagenesis and lead to cancer progression [24]. IL-6 is an important pro-inflammatory cytokine, with the ability to induce the expression of iNOS, which increases NO production [25]. In addition, the overproduction of reactive oxygen species (ROS) during inflammation can damage vital cellular components and cause oxidative stress [26]. Therefore, finding compounds that can suppress the aberrant production of inflammatory markers could potentially alleviate chronic inflammation and thereby prevent associated chronic diseases.

The current study aimed to extract sorghum bioactives from two novel sorghum genotypes using different extraction systems, including water or ethanol with or without HCl, and comparatively studied the phenolic contents and profiles, and measured the downstream biological activities (including the in vitro antioxidant properties and the anti-inflammatory capacities) in LPS-stimulated RAW 264.7 macrophages.

## 2. Materials and Methods

### 2.1. Materials

Novel sorghum black-seeded germplasms SC84 and PI570481 were used in this study. SC84 (converted line from PI 534144; origin: Uganda) is a high phenolic, photo-insensitive sorghum which readily grows in both Mexico and Kansas. SC84MX was grown in the Kansas State University (KSU) winter nursery in Mexico, while SC84KS was grown at the KSU Agricultural Research Center, Hays, Kansas. PI570481 (origin: Sudan), a photosensitive and non-producing grain in most areas of the United States, was grown at the KSU winter nursery in Mexico. The murine macrophage RAW 264.7 cell line was obtained from the American Type Culture Collection (Manassas, VA, USA). Growth media Dulbecco’s Modified Eagle Media (DMEM) 1X was purchased from Corning Inc. (Corning, NY, USA) and fetal bovine serum (FBS) was from Life Technologies (Carlsbad, CA, USA). The IL-6 ELISA kit was purchased from BioLegend (San Diego, CA, USA). All chemicals were purchased from either Sigma-Aldrich (St Louis, MO, USA) or Fisher Scientific (Atlanta, GA, USA) unless otherwise specified.

### 2.2. Sorghum Phenolics Extraction

Sorghum phenolics were extracted by four solvent systems: deionized (DI) water and ethanol, with or without 0.1% *v*/*v* HCl, using a 1:10 solid-to-liquid ratio (*w*/*v*). Briefly, whole grains of sorghum were ground and passed through a 30-mesh sieve. Approximately 30 g of each grain flour was suspended into 300 mL of extracting solvent and stirred continuously for 16 h at room temperature (21 °C) in the dark. The mixture was then centrifuged at 8000× *g* for 20 min at 4 °C and the supernatant was decanted. A 10 mL aliquot of each extract was stored at 4 °C in the dark until the phenolics were quantified. Ethanol extracts were concentrated by rotary evaporation under vacuum at 40 °C. The remaining liquid from the ethanol extracts and water extracts was freeze dried. Extraction was carried out for two independent replicates for each sorghum sample.

### 2.3. Quantification of Phenolic Extracts

#### 2.3.1. Measurement of Total Soluble Polyphenols

Total soluble polyphenols were quantified following a previous protocol with modifications [27]. Briefly, 10 μL of different phenolic extracts and gallic acid standards varying from 0 to 1 mg/mL were plated in triplicate in a 96-well plate. Samples and standards were mixed with 25 μL of 1 N Folin–Ciocalteu reagent and 25 μL of 20% sodium bicarbonate, followed by 150 μL DI water. The mixture was incubated for 30 min at room temperature before recording the absorbance at 630 nm using a Cambrex ELX 808 microplate reader (Biotek Instruments, Winooski, VT, USA). Total soluble polyphenols were calculated according to the gallic acid standard curve and expressed as mg gallic acid per gram sorghum. All measurements were performed as independent triplicates.

#### 2.3.2. Measurement of Total Flavonoids

Determination of total flavonoids was performed by the aluminum conjugation method as previously reported [28]. In a 96-well plate, 20 μL of phenolic extracts and quercetin standards were plated in triplicate, followed by the addition of 80 μL methanol and 100 μL 2% AlCl_3_·6H_2_O in methanol. The mixture was incubated for 30 min at room temperature and the absorbance was read at 405 nm using a Cambrex ELX 808 microplate reader. Total flavonoids were quantified using the quercetin standard curve and expressed as mg quercetin equivalent per gram sorghum. All experiments were performed as independent triplicates.

#### 2.3.3. Measurement of Total Tannins

Total tannins were analyzed using the modified HCl–vanillin assay as reported previously [29]. Twenty microliters of sorghum extract and epicatechin standards were loaded in triplicate into a 96-well plate, followed by 30 µL methanol and 150 µL of vanillin working reagent, which was prepared by mixing equal volumes of 1% vanillin solution and 8% *v*/*v* HCl in methanol. The plate was incubated for 10 min at room temperature and read at 490 nm using a Cambrex ELX 808 microplate reader. Total tannins were determined using the epicatechin standard curve, expressed as mg epicatechin per gram sorghum. All measurements were performed as independent triplicates.

#### 2.3.4. Measurement of Total Anthocyanins and 3-Deoxyanthocyanidins

Total 3-deoxyanthocyanidins and anthocyanins were measured using the spectrophotometric method reported previously with modifications [28]. The absorbance of 200 μL extracts plated in triplicate in a 96-well plate were read at 490, 520 and 700 nm, respectively, using a Synergy HT microplate reader (Biotek Instruments, Winooski, VT, USA). Total 3-deoxyanthocyanidins (T-3DA) were quantitatively determined using Equation (1) and expressed as μg luteolinidin per gram sorghum:

(1) $$T-3\mathrm{DA}\;(\mu g\;\mathrm{luteolinidin}/g\;\mathrm{sorghum})=\frac{\left(Abs_{490}-Abs_{700}\right)\times 271.24\times 1,000,000\times \left(vol\;of\;extract,\;L\right)}{35,000\times 0.45\times \left(wt\;of\;sorghum,\;g\right)}$$

Total anthocyanins (TA), reported as µg cyanidin-3-glucoside (C3G) per gram sorghum, were calculated using Equation (2):

(2) $$\mathrm{TA}\;(\mu g\;C3G/g\;\mathrm{sorghum})=\frac{\left(Abs_{520}-Abs_{700}\right)\times 449.38\times 1,000,000\times \left(vol\;of\;extract,\;L\right)}{26,900\times 0.45\times \left(wt\;of\;sorghum,\;g\right)}$$

where *Abs*_490_, *Abs*_520_, and *Abs*_700_ are absorbance at 490, 520 and 700 nm, respectively; 271.24 and 449.38 are the respective molecular weights of luteolinidin and C3G; 35,000 and 26,900 are the molar extinction coefficients of luteolinidin and C3G, respectively; 0.45 is a conversion factor from a conventional 1-cm pathlength method; vol, L is the volume of the extracting solvent in liters and wt, g is the weight of sorghum in grams.

### 2.4. HPLC Analysis of Phenolic Compounds

The identification of sorghum phenolic compounds was conducted according to a reported protocol with slight modifications [30], using an Agilent 1200 HPLC system (Agilent Technologies, Santa Clara, CA) equipped with a G1329A auto-sampler, a G1311A quaternary pump, a G1315D diode array detector, a G1322A degasser, and a G1316A column thermostat. Briefly, 20 mg lyophilized powder of sorghum extracts were suspended in 1 mL of DI water and sonicated for 15 min, followed by continuous vortexing for 60 min at room temperature. After centrifugation at 20,000 × *g* for 30 min at 4 °C, supernatants were filtered through 0.45 μm polyvinylidene (PVDF) membranes and 20 μL was injected to a Zorbax Eclipse C-18 column (4.6 × 150 mm, 5.0 μm; Agilent Technologies) to separate sorghum phenolics using the following conditions: the mobile phase was composed of 4% formic acid in water (solvent A) and acetonitrile (solvent B); the flow rate was 1.0 mL/min with a gradient system as follows: 0–20 min, 12–20% B; 20–40 min, 20–50% B; 40–50 min, 50% B; 50–52 min, 50–12%; and 52–55 min, 12% B. Column temperature was maintained at 35 °C. Commercial phenolic standards were dissolved in dimethyl sulfoxide and diluted in acetonitrile to a final concentration of 100 ppm, and were optimally detected either at 280 or 340 nm. The identification of phenolic compounds was determined based on the retention times of the standards.

### 2.5. Measurement of Antioxidant Activity

Freeze-dried sorghum extracts were dissolved in DI water (1:10 *w*/*v*), sonicated, vortexed and centrifuged as described in Section 2.4. The collected supernatants were sterile filtered using a 0.22 μm PVDF filter. The sterile-filtered supernatant was used for the evaluation of in vitro antioxidant and anti-inflammatory properties in the following assays.

The antioxidant activities of sorghum phenolic extracts were assessed by radical scavenging effects on nitric oxide (NO) radicals and 2,2-diphenyl-1-picrylhydrazyl (DPPH^•^) free radicals, and the evaluation of the oxygen radical absorbance capacity (ORAC). These methods were adapted from a previous paper [31].

#### 2.5.1. NO Scavenging Assay

In a 96-well plate, 50 μL of sample supernatants at concentrations of 50 and 100 μg gallic acid eq/mL and a control (DI water) were plated in triplicate, followed by adding 50 μL of DI water and 25 μL of 100 mM sodium nitroprusside. In a duplicate plate, 25 μL of DI water instead of sodium nitroprusside was mixed with samples and the control. Plates were incubated for 2 h at room temperature and 100 μL of Griess reagent containing an equal volume of 1% sulfanilic acid in 5% phosphoric acid and 0.1% *N*-(1-napthyl)-ethylenediamine dihydrochloride was subsequently added. After incubation for another 15 min at room temperature, absorbance was read at 550 nm using a Synergy HT microplate reader (Biotek Instruments). Results were presented as % nitric oxide with respective to the control after subtracting the absorbance of samples mixed with DI water. All experiments were performed as independent triplicates.

#### 2.5.2. DPPH Radical Scavenging Assay

One hundred microliters of sample supernatants (50 or 100 μg gallic acid eq/mL) and blank (DI water) were plated in triplicate in a 96-well plate, followed by adding 100 μL of 100 µM DPPH solution that was freshly prepared in methanol. The plate was incubated for 30 min in the dark at room temperature. In order to eliminate the color interference, a mixture of sample and methanol instead of DPPH solution were tested as reference at the same time. The absorbance was read at 517 nm using a Synergy HT microplate reader (Biotek Instruments), and the production of DPPH radicals was calculated as follows after subtracting the absorbance of the samples mixed with methanol. All measurements were performed as independent triplicates.

(3) $$\%\mathrm{DPPH}\;\mathrm{Production}\;=\;(\frac{∆Absorbance\;of\;sample}{∆Absorbance\;of\;blank})\;\times \;100$$

#### 2.5.3. ORAC Assay

Twenty-five microliters of Trolox standards (ranging from 3.125 to 100 μM), blank and sample supernatants (at 100 μg gallic acid eq/mL that were further diluted 10 times with phosphate buffer (pH 7.4)) were added in triplicate in a black 96-well plate, followed by mixing with 150 μL of fluorescein working solution (75 mM in phosphate buffer). The plate was incubated for 30 min in the dark at 37 °C before the addition of 25 µL 2,2’-azobis(2-amidonpropane) dihydrochloride (41.5 mg/mL in phosphate buffer). The fluorescence was read at 485 nm/20 nm excitation and 528 nm/ 20 nm emission every minute for 2 h at 37 °C using a Synergy microplate reader (BioTek, Winooski, VT, USA). The ORAC value was expressed as μmol Trolox equivalent per milligram sample calculated from the generated Trolox standard curve.

### 2.6. Anti-Inflammatory Activity in LPS-Induced RAW 264.7 Macrophages

#### 2.6.1. Cell Culture and Cell Proliferation

Murine RAW 264.7 macrophages were grown and maintained in DMEM (Corning Inc., Corning, NY, USA) supplemented with 10% heat-inactivated FBS (Life Tech, Carlsbad, CA, USA) and 1% *v*/*v* penicillin/streptomycin (Life Tech, Carlsbad, CA, USA) at 37 °C in a humidified incubator containing 5% CO_2_, and were sub-cultured every 2 days. Cells were seeded at 2.5 × 10^4^ cells/well in 96-well plates in 200 μL media and allowed to attach overnight. Cells were then treated with different sterile-filtered supernatants of sorghum extracts as prepared in Section 2.5 that were diluted with growth media to 50 or 100 μg gallic acid eq/mL for 24 h in the presence of 1 μg/mL LPS. The untreated cells and LPS only treated cells served as negative and positive controls, respectively. After treatment, the culture media was collected from each well for further analysis, and cells were studied with the CellTiter 96^®^ Aqueous One Solution Cell Proliferation Assay (Promega, Madison, WI, USA) to test viability by incubating with 100 μL plain DMEM containing 10% *v*/*v* MTS for 3 h, and reading the absorbance at 490 nm (Cambrex ELX 808 microplate reader, Biotek Instruments, Winooski, VT, USA). Cell proliferation was presented as the percentage relative to the absorbance of negative control cells using the following formula. All experiments were performed in at least three trials, with four replicates per trial.

(4) $$\mathrm{Cell}\;\mathrm{viability}\;(\%)\;=\;\frac{Abs_{treated}}{Abs_{untreated}}\;\times \;100$$

where $Abs_{treated}$ is the absorbance of wells with cells treated with LPS or sorghum samples, while $Abs_{untreated}$ is the absorbance of negative control cells.

#### 2.6.2. Measurement of Pro-Inflammatory Markers

The collected culture media were used to examine the production of NO and the pro-inflammatory cytokine IL-6. NO was detected using the Griess reagent assay. Briefly, 100 μL of cell conditioned media and sodium nitrite (0–100 μM) as standards were plated in triplicate in a 96-well plate, followed by adding 100 μL of Griess reagent. After 5 min incubation at room temperature, the absorbance was recorded at 550 nm (Synergy H1 Hybrid Multi-Mode Reader, BioTek Instruments, Inc., Winooski, VT, USA). The calculated nitrite using the sodium nitrite standard curve was used for NO production.

The secreted IL-6 in the cell-conditioned media was determined by enzyme-linked immunosorbent assay (ELISA) using a commercial ELISA Max^TM^ Deluxe kit following the manufacturer’s protocol (BioLegend, San Diego, CA, USA). Absorbance was read at 450 nm and concentrations of IL-6 were quantified via the generated standard curve.

#### 2.6.3. Measurement of Intracellular Reactive Oxygen Species

Intracellular reactive oxygen species (ROS) amounts were determined by fluorescence microscopy and spectrophotometry as previously reported with some modifications [32], such as using 2′,7′-dichlorofluorescein diacetate (DCFDA) which is oxidized to a fluorescent dichlorofluorescein by hydroxyl and peroxyl radicals [33].

*Fluorescent microscopy.* RAW 264.7 macrophages were seeded in a 48-well plates at 5 × 10^4^ cells/well in 500 μL growth DMEM overnight before being treated with different sterile-filtered supernatants of sorghum extracts at 100 μg gallic acid eq/mL for 24 h. Cells were stimulated with 1 μg/mL LPS for the last 18 h. After treatment, the spent media was removed, and cells were washed with 500 μL ice-cold PBS. After washing, cells were incubated with 200 μL PBS containing 10 μM DCFDA for 30 min in the dark at 37 °C in a 5% CO_2_ incubator. The dye solution was then removed, and cells were washed with ice-cold PBS twice. The cellular ROS was detected by observing fluorescence images using the green fluorescent protein channel of the EVOS microscope (Thermo Fisher Scientific, Waltham, MA, USA) with 10× magnification.

*Fluorescence spectrophotometry.* Cells were seeded as described in the cell proliferation section but in a black 96-well plate. After overnight attachment, cells were treated the same way as depicted in the fluorescent microscopy sections with sorghum extracts at 50 and 100 μg gallic acid eq/mL. Afterwards, cells were washed with 200 μL ice-cold PBS followed by incubation with 200 μL of 10 μM DCFDA solution in PBS for 30 min at 37 °C in an incubator with 5% CO_2_. The ROS levels in cells were quantified by recording the fluorescence intensity at excitation and emission wavelengths of 485 nm and 528 nm, respectively, using a Synergy H1 Hybrid Multi-Mode Reader (BioTek Instruments, Inc., Winooski, VT, USA).

### 2.7. Statistical Analysis

All experiments were performed in three independent trials and results were shown as mean ± standard deviation. Data were analyzed using one-way ANOVA by the IBM SPSS Statistics version 25.0 software (SPSS Inc., Chicago, IL, USA). Tukey’s test was used for mean comparisons and *p* < 0.05 was considered significantly different. The Pearson correlation coefficient was determined using the Bivariate process of SPSS.

## 3. Results

### 3.1. Sorghum Phenolic Contents in Different Extracting Systems

The concentrations of biologically active compounds from the studied three novel sorghum samples in different extracting solvents are shown in Table 1. As shown, the content of sorghum phenolics varied among different extraction systems and sorghum samples. All ethanol extracts contained higher concentrations of bioactives than their aqueous counterparts, except for the total 3-deoxyanthocyanidins and anthocyanins showing the opposite tendency in SC84MX and SC84KS in the absence of HCl.

Total polyphenols both in water and ethanol extracts were enhanced by the addition of 0.1% *v*/*v* HCl, with SC84MX and SC84KS sorghum samples being found to contain significantly higher contents than PI570481. Similarly, the acidified ethanol extracts had the highest concentrations of total flavonoids in contrast with other extraction systems. However, the acidified aqueous system received the lowest flavonoid content, containing only 0.59–0.79 mg quercetin eq/g sorghum grain among the different sorghum samples. On the other hand, the addition of HCl considerably lowered the levels of total tannins in both water and ethanol extracts. A significantly higher content of tannins was present for SC84MX and SC84KS in extracting solvents in the absence of HCl, whereas no significant differences in these three sorghum samples were found in the acidified extraction systems.

Regarding 3-deoxyanthocyanidins, HCl played a positive role in ethanolic solvents but a negative role in aqueous systems. For water extracts, compared to the other two sorghum samples, SC84MX had statistically higher values of 3-deoxyanthocyanidins, while in the acidified aqueous system, SC84KS possessed relatively higher concentration. However, in ethanolic systems, PI570481 had the highest concentration of 3-deoxyanthocyanidin when compared to SC84MX and SC84KS, which was greatly enhanced in the presence of HCl. The total anthocyanin levels followed the same pattern as 3-deoxyanthocyanidins, with SC84MX showing the highest levels among water extracts and PI570481 having the most abundant content in ethanolic systems.

**Figure 1:**
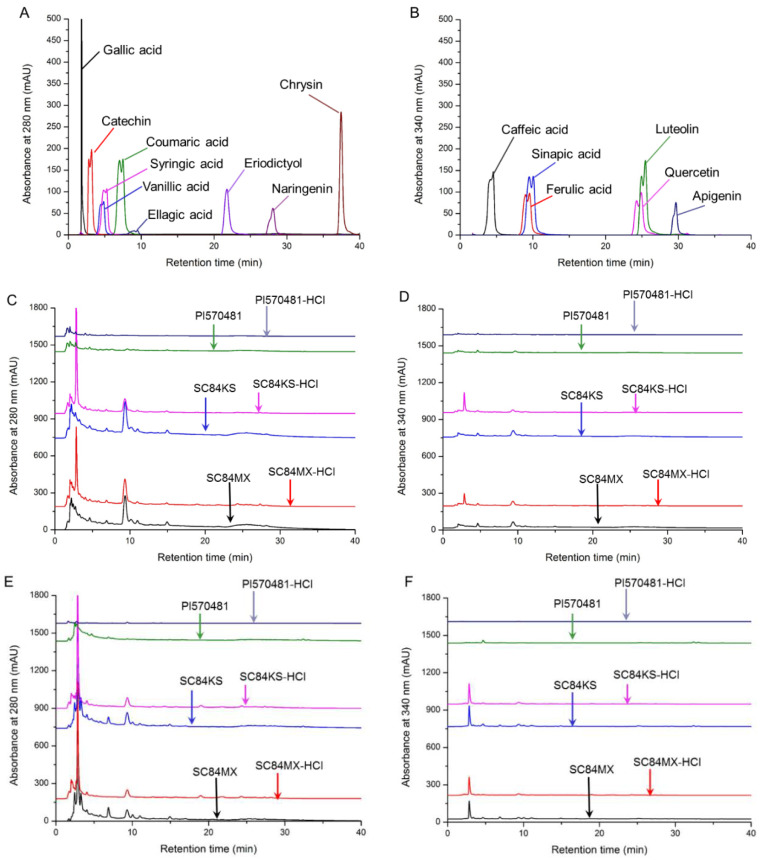
HPLC analysis of phenolic compounds in sorghum extracts. HPLC chromatograms of different phenolic standards detected at 280 nm (**A**) and 340 nm (**B**); HPLC chromatograms of sorghum water extracts detected at 280 nm (**C**) and 340 nm (**D**); HPLC chromatograms of sorghum ethanol extracts detected at 280 nm (**E**) and 340 nm (**F**).

HPLC was used to further study the sorghum phenolic compositions among the different extracting solvents and sorghum samples. Figure 1 shows HPLC profiles of sorghum phenolic extracts recorded at 280 nm and 340 nm. In accordance with the retention time of commercially available phenolic standards (Figure 1A,B), a variety of bioactive compounds were identified in different sorghum extracts, most of which were more prominent under the detection wavelength of 280 nm (Figure 1C–F). As shown in Table 2, SC84MX had a generally higher diversity of phenolic compounds than the other two sorghum samples, while PI570481 contained the least diversity, which may be responsible for the lower levels of total polyphenols, total flavonoids, and total tannins in PI570481 detected in the above spectrophotometric assays. However, 3-deoxyanthocyanidin was not determined in the HPLC analysis, which was largely quantified in PI570481, and may account for its potential biological activities. The differences in these bioactive constituents between sorghum samples and extracting solvents could possibly affect the potential bioactivities of these sorghum samples.

### 3.2. Sorghum Extracts Exert Antioxidant Activity

**Figure 2:**
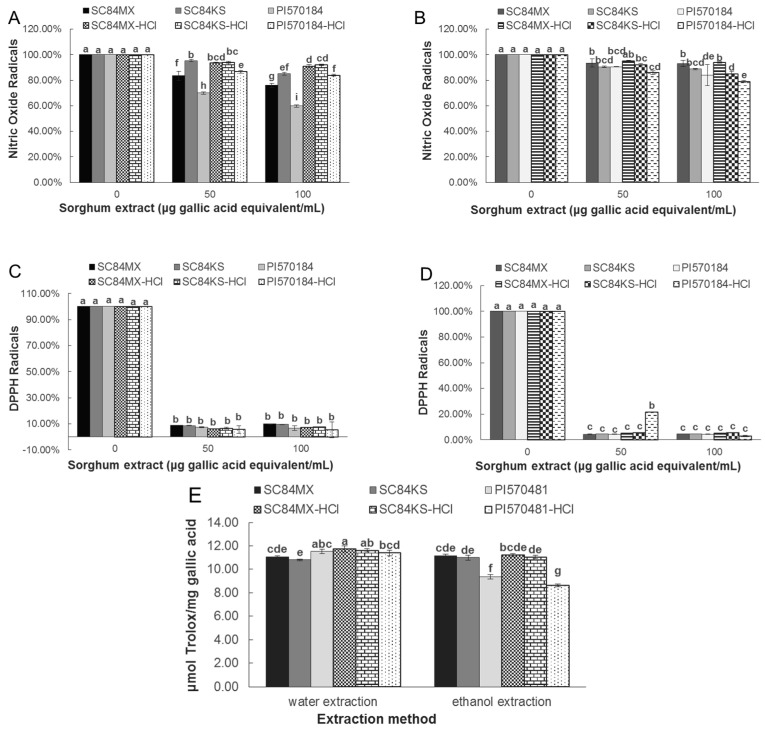
Antioxidant properties of sorghum phenolics extracts. Nitric oxide (NO) scavenging activity of (**A**) water extracts and (**B**) ethanol extracts; 2,2-diphenyl-1-picrylhydrazyl (DPPH) scavenging activity of (**C**) water extracts and (**D**) ethanol extracts; (**E**) oxygen radical absorbance capacity (ORAC) values of water and ethanol extracts. The percentage production of NO and DPPH were calculated with respect to the control. Error bars are standard deviations (*n* ≥ 3). Different letters a–i above columns indicate significant differences (*p* < 0.05).

The antioxidant properties of different sorghum extracts were evaluated by measuring their ability to scavenge DPPH and NO radicals, and by ORAC assay. As shown in Figure 2A, for the water extraction system, the absence of HCl favored the scavenging ability of sorghum extracts on NO radicals. Aqueous extracts of SC84MX inhibited NO production from 16.49% to 24.08% at 50 and 100 μg gallic acid eq/mL, respectively, while SC84KS inhibited it from 4.61% to 15.06%. PI570481 exerted the strongest inhibition activity, ranging from 30.07% to 40.10% at the same respective concentration. Meanwhile, the corresponding inhibitory activity declined to 8.93%, 7.82%, and 16.09% for SC84MX, SC84KS, and PI570481, respectively, at 100 μg gallic acid eq/mL in the acidified aqueous system. Ethanol extracts of PI570481 exerted a better NO scavenging ability as well; however, it was slightly enhanced from 15.88% to 20.93% in the presence of HCl at 100 μg gallic acid eq/mL (Figure 2B), which did not happen with the other two sorghum samples. In comparison, vitamin C and gallic acid at 100 µg/mL inhibited NO radicals by 15.33% and 25.29%, respectively.

On the other hand, extracts were found to be more potent at DPPH radical scavenging than NO radical scavenging. In general, all extracts inhibited more than 90% of DPPH radicals, except for the 50 μg gallic acid eq/mL acidified ethanol extract of PI570481 (Figure 2C,D). This is similar to vitamin C and gallic acid DPPH activity, which inhibited DPPH radical production by 96% and 95%, respectively. In regard to the ORAC assay, the addition of HCl was recorded to slightly improve the ORAC value of the aqueous extracts of SC84MX and SC84KS, from 11.08 to 11.75 μmol Trolox/mg gallic acid and 10.81 to 11.62 μmol Trolox/mg gallic acid, respectively. The lowest ORAC value was found in the ethanolic extracts of PI570481, with a significant decrease from 9.35 to 8.64 μmol Trolox/mg gallic acid as affected by the addition of HCl. Ethanol extracts of SC84MX and SC84KS were not significantly influenced by HCl in terms of ORAC value.

### 3.3. Sorghum Extracts Alleviate LPS-Induced Inflammation in RAW 264.7 Macrophages

The potential anti-inflammatory properties of sorghum extracts were then evaluated using RAW 264.7 macrophages stimulated with LPS. Cell viability was initially investigated by the simultaneous addition of LPS and different sorghum extracts at 50 and 100 µg gallic acid eq/mL for 24 h; it was found to be at least 80% in the LPS-treated cells and was not significantly affected by the sorghum extracts at up to 100 µg gallic acid eq/mL (data not shown here, *p* > 0.05), suggesting non-toxicity. The anti-inflammatory activity of sorghum phenolic extracts was subsequently assessed by measuring their effects on the production of various pro-inflammatory molecules including NO, IL-6, and intracellular ROS.

**Figure 3:**
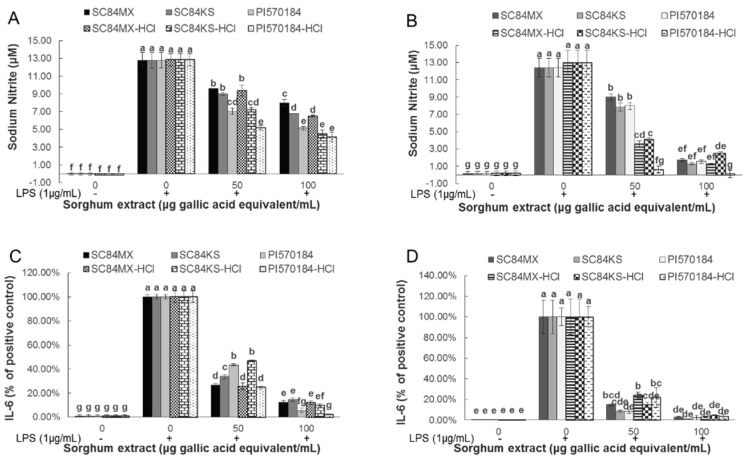
Effects of sorghum phenolic extracts on NO and interleukin (IL)-6 production in LPS-induced RAW 264.7 macrophages. NO production by macrophages as affected by LPS and sorghum (**A**) water extracts and (**B**) ethanol extracts; IL-6 production as affected by LPS and sorghum (**C**) water and (**D**) ethanol extracts. Percentage production of IL-6 was calculated with respect to the positive control. Error bars are standard deviations (*n* ≥ 3). Different letters above columns indicate significant differences (*p* < 0.05).

As shown in Figure 3A,B, LPS treatment led to a significant increase in the production of NO and this was dose-dependently counteracted by sorghum phenolic extracts at 50 and 100 µg gallic acid eq/mL treatments. For the aqueous extraction system, the NO inhibition ability of all sorghum extracts was enhanced by the addition of HCl. At 100 μg gallic acid eq/mL, water extracts of SC84MX, SC84KS, and PI570481 reduced the production of NO by 37.47%, 47.10%, and 59.80%, respectively, whereas in acidified aqueous extracts, the corresponding reductions were increased to 49.55%, 64.74%, and 67.76% (Figure 3A). Ethanol extracts generally exerted better capabilities in inhibiting NO production than water extracts. As for the ethanolic extraction system, compared to the absolute ethanol extracts, acidified ethanol extracts inhibited the production of NO to a much higher extent, enhanced from 27.45% to 72.45%, 37.02% to 68.32%, and 35.74% to 95.36% for SC84MX, SC84KS, and PI570481, respectively, for the 50 µg gallic acid eq/mL treatment. However, the 100 µg gallic acid eq/mL treatment of ethanolic extracts did not show such a significant difference, which could be attributed to low NO concentrations (Figure 3B).

Figure 3C,D show the differential abilities of the tested extracts to modulate the secretion of IL-6. As shown, LPS treatment led to a significant increase in the secretion of IL-6. The inhibition of IL-6 production at 100 µg gallic acid eq/mL treatment did not show significant differences between extracting solvents before and after the addition of HCl, but differentiate among sorghum samples, (especially in water extracts), ranging from 85.45% to 97.69% with PI570481 exerting the best activity. Meanwhile, ethanol extracts of sorghum samples exhibited a higher inhibitory ability of IL-6 than their water extract counterparts at 100 µg gallic acid eq/mL (*p* < 0.001 between each counterparts), while this observation was not found in PI570481 extracts with HCl (*p* = 0.222).

**Figure 4:**
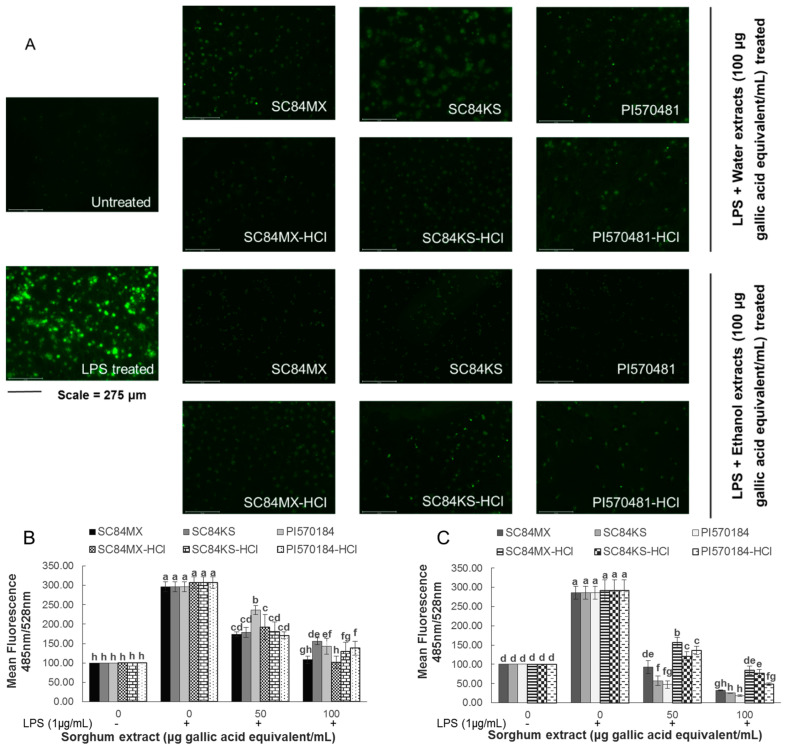
(**A**) Fluorescence microscopy analysis of reactive oxygen species (ROS) production in LPS-induced macrophages as affected by treatment with sorghum water and ethanol extracts (100 μg gallic acid equivalent/mL), pictures were taken at 10× magnification with a scale bar of 275 μm. Mean fluorescence values of ROS that were affected by sorghum (**B**) water and (**C**) ethanol extracts in LPS-induced macrophages were quantified by fluorescence spectrophotometry and were expressed with respect to the negative control. Error bars are standard deviations (*n* ≥ 3). Different letters above columns indicate significant differences (*p* < 0.05).

Intracellular ROS was also detected after the treatment with sorghum extracts and LPS using fluorescence microscopy and spectrophotometry. As shown in Figure 4A, compared to the untreated cells, significantly increased green fluorescence was observed after treatment with LPS, indicating the production of ROS. However, the strong fluorescence signals were greatly suppressed in the presence of different sorghum extracts at 100 µg gallic acid eq/mL. This observation was further validated by quantitative fluorescence spectrophotometry. As shown in Figure 4B,C, ethanol extracts in general exerted a better inhibition ability than water extracts. Moreover, absolute ethanol extracts of all sorghum samples have a better suppressive effect compared with their acidified ethanol extracts, enhanced from 71.14% to 88.72%, 73.60% to 91.05%, and 83.34% to 93.47% for SC84MX, SC84KS, and PI570481, respectively, at 100 µg gallic acid eq/mL. The same enhancement was not observed in the water extracts.

## 4. Discussion

As one of the most important leading crops in the world, sorghum has attracted increasing attention recently, which is driven by the potential health benefits associated with its components, especially the various phenolic compounds. Sorghum is a rich source of diverse phenolic compounds such as phenolic acids, flavonoids, tannins, 3-deoxyanthocyanidins and anthocyanins [1,2]. A handful of studies have demonstrated the health-promoting benefits of sorghum, including anti-inflammatory, anti-proliferative, and antioxidant properties [8,10,11,15]. The current study investigated the phenolic content and profiles of two novel sorghum genotypes and two separate growing environments for SC84 using different extraction systems, and subsequently evaluated the downstream biological activities regarding the in vitro antioxidant properties and the anti-inflammatory activities in LPS-induced RAW 264.7 macrophages.

Based on the spectrophotometric assays, the obtained content of sorghum phenolic compounds varied dramatically among sorghum samples and different extraction systems. Indeed, compositions of extraction systems play an important role in the content and yield of phenolic extracts from plant materials. Different solvent types with different polarities affect the extraction efficiency of phenolics. Phenolic compounds are often extracted in higher amounts in more polar solvents [34]. However, the solubility of phenolics is governed not only by the polarity of solvents, but also by the chemical nature of the phenolic compounds [35]. Higher concentrations of phytochemicals were reported to be present in ethanolic rather than methanolic extracts for 10 sorghum genotypes [28]. An effectiveness order of solvent types (methanol > aqueous ≥ ethanol ≥ acetone) was constructed for phenolic extracts from *B. buceras* and *P. californicum* [34]. In another study, the highest levels of phenolics were extracted from florets of sunflowers using 90% aqueous methanol [36]. The current study showed higher yields of total polyphenols, total flavonoids, and total tannins in ethanolic extracts than aqueous extracts over the three sorghum samples. In particular, ethanol was significantly more effective than water at extracting tannins, which was probably attributed to the polar-protic property of ethanol that could provide OH ions and make it easier to interact with the polar functional groups on tannins [37]. In contrast, water extraction was found to be more effective in terms of total 3-deoxyanthocyanidins and total anthocyanins. These differences could be due to the different physicochemical properties of the phenolic components.

Apart from solvent types, interfering substances in the extracting solvent is another key contributor to the final yield and content of phenolics in the extracts [38]. HCl was used in this study with the aim of lowering the pH value of the extraction system, thus altering the extract profiles. It was found that the addition of acid significantly enhanced the levels of total polyphenols in the ethanolic extracts for all sorghum samples studied; meanwhile, the acidified aqueous system was also found to be more effective than neutral water to extract total polyphenols from PI570481. Similar results were also reported by Shelembe et al.; under acidic conditions of water (pH 2), total phenolic compounds were increased from 5.8 to 6.7 mg catechin eq/g sorghum bran [39]. It is generally known that phenolic compounds in sorghum frequently exist in bound forms, which are mostly bound to arabinoxylan chains or lignin via covalent bonds [1]. Thus, the improved extractability was believed to be attributed to the breakage of the covalent bonds under acidic conditions and the subsequent release of the bound phenolic compounds which account for a significant proportion in cereals [39]. In addition, flavonoid glycosides are quite unstable in acidic environments and are easily hydrolyzed to aglycones [40]. It was reported that flavonoid aglycones have been identified and quantified by reversed-phase high-performance liquid chromatography (RP-HPLC) by acidic hydrolysis of the glycosidic residues bound to the flavonoid nuclei in 20 dry herbal samples [41]. Besides, acidified methanol and ethanol were commonly-used solvents to extract anthocyanins. The acid in these solvent systems was able to denature and rupture cell membranes and thus release anthocyanins [42]. Hence, it was not surprising to find considerably higher levels of total flavonoids, total 3-deoxyanthocyanidins and total anthocyanins in the acidified ethanolic extracts than their absolute ethanolic products in the current studied sorghum samples. However, excess addition and harsh acids need to be avoided, which may break down the innate structures of phenolics. At the same time, polyphenols contain abundant hydroxyl groups, which are highly susceptible to oxidation [1,2]. In this regard, the extraction efficiency is a conflicting consequence of the release of bound phenolics and the degradation of free and released polyphenols by excessive hydrolysis and oxidation. The effects of acid differ in diverse solvent types, and the different intrinsic chemical properties of phenolic compounds may contribute to the inconsistent effects of HCl found in water and ethanol systems in terms of the total flavonoids, total 3-deoxyanthocyanidins and total anthocyanins, as well as the decreased levels of tannins in both acidified aqueous and ethanolic extracts. Moreover, growth and environmental variations are believed to play important roles in different phenolic contents among sorghum samples [43]. The concentrations of obtained phenolic compounds among different extraction systems and sorghum types in this study were generally in accordance with some previous reports [28,30,44]. However, different extraction times, temperatures, sample-to-solvent ratios, different agitation methods and the number of replicate extractions can influence the extraction efficiency [45]. Variations in the genotypes of the sorghum may also contribute to the difference in phenolic contents among different studies.

In addition, a diverse range of phenolic compounds were identified by HPLC regarding the designed extraction systems and the studied sorghum types. Acid treatment did not result in a significant increase of diversity among different sorghum samples, but it did affect the phenolic compositions. For instance, the acidified ethanol treatment of SC84MX and SC84KS samples enhanced the extraction of flavanones such as eriodictyol and naringenin, but suppressed the extraction of catechin, sinapic acid and luteolin compared to the ethanolic counterpart. Agbangnan et al. reported different families of phenolic compounds extracted by 25% aqueous ethanol and water under different pH [46]. The lower diversity of polyphenols in PI570481 extracts was possibly due to unexpected loss during the redissolution when samples were prepared for HPLC analysis, as well as the lack of 3-deoxyanthocyanidin detection, which presented significantly higher levels in ethanol extracts of PI570481 as shown by the spectrophotometric assays. Additionally, genetic variations are important factors that can also be responsible for different phenolic profiles among sorghum samples [43].

Given the various phenolic compounds in sorghum extracts, we investigated the antioxidant activity of the obtained sorghum products. It is well believed that the potential role of phenolic compounds in preventing human diseases is partially ascribed to their antiradical activity by donating hydrogen atoms from the aromatic hydroxyl groups to free radicals [47]. Free radicals such as superoxide radical (O_2_^-^), hydroxyl radical (OH^-^), peroxyl radical (ROO) and nitric oxide radical (NO) play a vital role in biological metabolism [48]. However, the imbalance of free radicals and antioxidants, in terms of oxidative stress, is the leading cause of various chronic diseases [18]. Sorghum extracts have been well documented for their significant antioxidant capacity due to their ample polyphenols [49]. In the current study, it was found that the NO scavenging activity of sorghum extracts varied greatly from 4.83% to 40.10% in a concentration-dependent manner among the different extraction systems and sorghum types. Meanwhile, the ability to inhibit DPPH production appeared to be significantly more potent, as all sorghum extracts quenched more than 90% of DPPH radicals except the lower concentration treatment of PI570481 extracts in acidified ethanol, which was comparable to another study where an average of 90% antioxidant activity against DPPH was reported for three different genotype sorghum flours [50]. The different extents of radical scavenging capacity based on different radicals is probably due to variations in the phenolic profiles and different stabilities of these radicals in solutions. Radicals in DPPH are stabilized by a single bond between two nitrogen atoms, while in NO, they are stabilized by a double bond between nitrogen and oxygen atoms [31]. In this light, sorghum extracts in this study are possibly better antioxidants against less stable radicals. On the other hand, the ORAC assay is based on the delay of oxidation. It measures the ability of antioxidants to protect proteins from damage by free radicals [51]. The ORAC values of different sorghum extracts obtained in this study ranged from 8.64 to 11.75 μmol Trolox/mg sorghum grain, which were higher than those previously found [52], suggesting the extraction systems used in this study were efficient at extracting potent antioxidant compounds.

It is worth mentioning that the DPPH radical activity of sorghum extracts obtained in this study was found to be positively correlated to the content of total tannins (Table 3). Previous studies have highlighted the high antioxidant activity of condensed tannins in sorghum, which are not common in other major cereals. Dykes et al. attributed that the higher antioxidant activity of sorghum hybrids with a pigmented testa compared to other sorghum types mainly came from condensed tannins [53]. In another study, in vitro antioxidant properties were found to be strongly correlated to the condensed tannins detected in the investigated sorghums (r > 0.96, *p* < 0.01) [54]. Hagerman et al. reported that tannins were 15–30 times more effective than simple phenolics at radical scavenging, which may be largely attributed to their proximity to many aromatic rings and hydroxyl groups, as well as the fact that tannins had little or no pro-oxidant activity [15]. In short, the studied sorghum extracts are better antioxidants against less stable radicals, which is positively correlated to the total tannins, and these extracts are strong antioxidants in delaying oxidation processes.

Inspired by the strong antioxidant capacity, the anti-inflammatory activity of the obtained sorghum extracts was subsequently studied. As stated earlier, oxidative stress is implicated in various health conditions such as chronic inflammation, which could consequently result in various chronic diseases including carcinogenesis and cardiovascular diseases [18,22]. A number of pro-inflammatory molecules are generated during inflammation, such as pro-inflammatory cytokines, chemokines, transcription factors, enzymes and reactive oxygen or nitrogen species [19]. In this sense, the attenuation of inflammation in terms of the reduction of pro-inflammatory markers is believed to be directly related to disease prevention. Sorghum bioactives, acting individually or in complex extracts, have been well recognized to have anti-inflammatory properties both in vitro and in vivo. For instance, isolated benzoic and cinnamic acid derivatives from sorghum grains were reported to inhibit the production of NO in LPS-induced RAW 264.7 macrophages with an associated reduction in the expression of iNOS and cyclooxygenase-2 [55]. Burdette et al. demonstrated the dose-dependent inhibitory activity of black sorghum bran (non-tannin) extracts on the secretion of tumor necrosis factor-α and IL-1β in LPS-activated human mononuclear cells in vitro [56]. In addition, ethanolic extracts from both sumac (tannin) and black (non-tannin) sorghum brans were capable of reducing inflammation induced by 12-O-tetradecanoylphorbol acetate in rats [56]. In contrast, neither wheat nor rice brans showed the same anti-inflammatory properties in those two studies [56]. In another study, sorghum flour of various genotypes differing in phenolic compositions were reported to reduce low-grade inflammation and oxidative stress in adult Wistar rats when added as 21–26% of the diet without altering jejunum morphology [50].

In the current study, the effectiveness of sorghum extracts against inflammation was confirmed by the potent inhibitory activity of the production of NO, IL-6 and ROS by LPS-induced RAW 264.7 macrophages. The selected sorghum extracts were found to inhibit the production of these pro-inflammatory molecules, with higher inhibition exhibited by ethanolic extracts. On the other hand, the suppressive activity of sorghum extracts on the production of NO, IL-6 and ROS in this study was found to positively correlate to the total tannins rather than other phenolic compounds, which is consistent with the DPPH results (Table 3). Such positive associations can be attributed to the most dominant presence of tannins compared to other phenolic compounds in the studied sorghum extracts. It was reported that compared to other sorghum genotypes, sorghum extracts rich in tannins had greater inhibitory activity against hyaluronidase, an important enzyme associated with inflammation [57]. This superior inhibitory effect was believed to be attributed to the ability of the tannins to complex the enzyme through competitive binding [57]. In another study, red rice extracts rich in proanthocyanidin demonstrated anti-inflammatory activity via the suppression of activator protein-1 and nuclear factor-κB pathways in LPS-activated RAW 264.7 macrophages [58].

## 5. Conclusions

In summary, the current study comparatively investigated the phenolic contents of three novel sorghum extracts, SC84MX, SC84KS and PI570481, that were obtained by aqueous and ethanolic extraction systems in the absence or presence of 0.1% *v*/*v* HCl, and tested their downstream antioxidant and anti-inflammatory activities. The results suggest that no single extraction system could effectively extract all phenolic compounds. The extraction efficiency is a balancing consequence of solvent polarities and the chemical nature of bioactive compounds, as well as the release of bound phenolics and the degradation caused by excessive hydrolysis and oxidation under acidic conditions. The obtained sorghum extracts exerted dose-dependent scavenging activity on NO and DPPH radicals, with a higher inhibitory extent found in the DPPH assay. Under non-toxic concentrations, the selected sorghum extracts possessed potent anti-inflammatory properties in LPS-induced RAW 264.7 macrophages, associated with the inhibition of NO, IL-6 and ROS production. Such health-promoting capacities may be attributed to the most dominant presence of tannins in the obtained sorghum extracts, since the tannin content was found to be positively correlated to the DPPH scavenging activity, as well as the inhibitory effects on pro-inflammatory markers. In conclusion, this study contributes to the growing body of evidence that sorghum extracts, abundant in phenolic compounds, may benefit human health. Follow-up studies that isolate and test individual constituents in the extracts and different combination studies may help to determine the specific health protective effects of each constituent, and thus provide clues for specialty breeding of sorghum for health promotion.

## Figures and Tables

**Table 1:** Different contents of phenolic compounds as measured by spectroscopy in novel sorghum samples using different extraction systems.

Sorghum Sample	Extract without HCl		Extract with 0.1% *v*/*v* HCl	
	Water Extraction	Ethanol Extraction	Water Extraction	Ethanol Extraction
**Total Polyphenols, mg gallic acid eq/g**				
SC84MX	8.55 ± 0.07 ^f^	9.58 ± 0.52 ^de^	9.00 ± 0.2 ^ef^	18.26 ± 0.79 ^b^
SC84KS	8.23 ± 0.44 ^f^	10.24 ± 0.25 ^d^	8.50 ± 0.11 ^f^	19.60 ± 0.88 ^a^
PI570481	1.42 ± 0.05 ^i^	6.02 ± 0.33 ^g^	3.24 ± 0.13 ^h^	12.61 ± 1.07 ^c^
**Total Flavonoids, mg quercetin eq/g**				
SC84MX	1.13 ± 0.02 ^cd^	1.29 ± 0.32 ^bc^	0.79 ± 0.06 ^e^	1.74 ± 0.01 ^a^
SC84KS	0.89 ± 0.04 ^de^	1.18 ± 0.20 ^cd^	0.72 ± 0.02 ^e^	1.65 ± 0.03 ^a^
PI570481	0.57 ± 0.08 ^e^	0.64 ± 0.08 ^e^	0.59 ± 0.08 ^e^	1.55 ± 0.01 ^ab^
**Total Tannins, mg epicatechin/g**				
SC84MX	21.96 ± 0.64 ^d^	131.11 ± 8.39 ^a^	5.28 ± 0.24 ^e^	66.06 ± 3.78 ^c^
SC84KS	21.22 ± 1.11 ^d^	136.11 ± 12.06 ^a^	6.17 ± 0.08 ^e^	58.82 ± 0.37 ^c^
PI570481	11.11 ± 1.92 ^e^	99.44 ± 10.72 ^b^	6.03 ± 1.16 ^e^	62.80 ± 3.59 ^c^
**Total 3-deoxyanthocyanidins, μg luteolinidin eq/g**				
SC84MX	127.40 ± 2.04 ^b^	29.72 ± 0.92 ^g^	28.40 ± 3.25 ^g^	92.54 ± 1.07 ^d^
SC84KS	97.93 ± 1.08 ^d^	35.82 ± 0.40 ^f^	35.64 ± 6.51 ^f^	112.63 ± 1.55 ^c^
PI570481	18.31 ± 0.89 ^h^	68.37 ± 0.15 ^e^	16.86 ± 1.82 ^h^	325.23 ± 4.76 ^a^
**Total Anthocyanins, μg cyanidin-3-glucoside eq/g**				
SC84MX	168.05 ± 1.66 ^b^	48.14 ± 0.77 ^g^	34.55 ± 3.60 ^h^	104.66 ± 1.46 ^e^
SC84KS	126.88 ± 1.41 ^d^	64.49 ± 1.45 ^f^	48.49 ± 8.15 ^g^	126.27 ± 2.77 ^d^
PI570481	38.86 ± 1.61 ^h^	149.24 ± 0.45 ^c^	26.80 ± 0.73 ^i^	554.87 ± 7.38 ^a^

Different letters within the same bioactive indicate significant differences (*p* < 0.05).

**Table 2:** Identification of flavones and flavanones in extracts of novel sorghum samples.

Standards	WL (nm)	RT (min)	Sample Area											
			Water Extracts		Ethanol Extracts		Water Extracts		Ethanol Extracts		Water Extracts		Ethanol Extracts	
			MX	MX-HCl	MX	MX-HCl	KS	KS-HCl	KS	KS-HCl	PI	PI-HCl	PI	PI-HCl
Gallic acid	280	1.81	1346.4	1690.5	226.7	964.9	1527.7	1737.3	321.7	825.2	1517.4	1413.8	282.4	**261.3**
Catechin	280	3.17	2963.5	2143.5	5557.2	N/D	7842.0	N/D	6032.3	N/D	N/D	N/D	N/D	N/D
Vanillic acid	280	4.77	2816.0	1935.0	2157.0	726.0	4154.4	1451.3	3181.8	861.2	718.4	404.7	3846.2	N/D
Syringic acid	280	4.90	1101.0	986.0	N/D	N/D	N/D	540.9	N/D	N/D	N/D	131.6	N/D	N/D
Coumaric acid	280	7.03	2507.0	N/D	3658.4	248.2	2701.9	1923.6	3994.5	322.8	497.4	179.2	1631.5	N/D
Ellagic acid	280	9.46	8201.7	8110.6	3200.5	1616.5	8974.0	3549.6	4303.9	2148.3	N/D	N/D	386.6	**128.1**
Eriodictyol	280	21.75	139.5	290.3	N/D	423.0	116.4	N/D	N/D	169.0	N/D	N/D	N/D	N/D
Naringenin	280	28.08	1108.6	264.0	N/D	284.3	929.9	N/D	N/D	271.0	N/D	N/D	N/D	N/D
Chrysin	280	37.45	N/D	N/D	N/D	N/D	N/D	N/D	N/D	N/D	N/D	N/D	N/D	N/D
Caffeic acid	340	4.47	594.7	483.8	262.2	138.2	565.8	507.3	437.2	242.4	396.0	159.2	324.5	N/D
Ferulic acid	340	9.35	1239.1	1081.2	301.9	365.7	1448.4	506.9	468.6	458.8	N/D	N/D	N/D	N/D
Sinapic acid	340	9.89	302.6	N/D	195.9	N/D	N/D	177.4	182.7	N/D	358.2	115.6	N/D	N/D
Quercetin	340	24.88	N/D	**56.9**	N/D	N/D	N/D	N/D	N/D	N/D	N/D	N/D	N/D	N/D
Luteolin	340	25.45	N/D	N/D	**106.0**	N/D	N/D	N/D	**128.5**	N/D	N/D	N/D	**91.2**	N/D
Apigenin	340	29.65	N/D	N/D	N/D	N/D	N/D	N/D	N/D	N/D	N/D	N/D	N/D	N/D

MX: SC84MX; KS: SC84KS; PI: PI570481; MX-HCl: extract solvent contains 0.1% *v*/*v* HCl; WL: wavelength; RT: retention time; N/D: not detected.

**Table 3:** Pearson correlation, r, among bioactive concentrations, antioxidant capacity and inhibition of pro-inflammatory markers of sorghum extracts.

Parameter	Parameter										
	TP	TF	TT	T3DA	TA	NO	DPPH	ORAC	RAW-NO	IL-6	ROS
TP	1	0.911 ** (<0.0001)	0.313 (0.323)	0.420 (0.174)	0.289 (0.363)	−0.496 (0.101)	0.227 (0.478)	−0.123 (0.703)	0.430 (0.163)	0.128 (0.692)	0.337 (0.284)
TF		1	0.443 (0.149)	0.563 (0.057)	0.452 (0.140)	−0.315 (0.318)	0.339 (0.281)	−0.248 (0.437)	0.514 (0.087)	0.257 (0.420)	0.415 (0.180)
TT			1	0.059 (0.856)	0.114 (0.724)	−0.341 (0.279)	0.631 * (0.028)	−0.379 (0.224)	0.730 ** (0.007)	0.649 * (0.022)	0.890 ** (<0.0001)
T3DA				1	0.980 ** (<0.0001)	0.149 (0.643)	0.253 (0.428)	−0.774 ** (0.003)	0.313 (0.322)	−0.005 (0.987)	0.221 (0.491)
TA					1	0.188 (0.558)	0.353 (0.260)	−0.855 ** (<0.0001)	0.378 (0.225)	0.080 (0.806)	0.298 (0.348)
NO						1	−0.163 (0.613)	−0.126 (0.693)	−0.289 (0.362)	−0.066 (0.839)	−0.342 (0.277)
DPPH							1	−0.518 (0.085)	0.937 ** (<0.0001)	0.896 ** (<0.0001)	0.724 ** (0.008)
ORAC								1	−0.541 (0.070)	−0.301 (0.342)	−0.539 (0.071)
RAW-NO									1	0.862 ** (<0.0001)	0.750 ** (0.005)
IL-6										1	0.630 * (0.028)
ROS											1

TP: total polyphenols; TF: total flavonoids; TT: total tannins; T3DA: total 3-deoxyanthocyanidins; TA: total anthocyanins; NO: nitric oxide scavenging activity; DPPH: 2,2-diphenyl-1-picrylhydrazyl scavenging activity; ORAC: oxygen radical absorbance capacity; RAW-NO: nitric oxide inhibition activity in RAW 264.7 macrophages; IL-6: interleukin-6; ROS: intracellular reactive oxygen species. Number in parenthesis signifies *p*-value. * Correlation is significant at 0.05 level (2-tailed). ** Correlation is significant at 0.01 level (2-tailed).
